# New Insights Into the Role of Follicle-Stimulating Hormone in Sex Differentiation of the Protogynous Orange-Spotted Grouper, *Epinephelus coioides*

**DOI:** 10.3389/fendo.2019.00304

**Published:** 2019-05-15

**Authors:** Minwei Huang, Jiaxing Chen, Yun Liu, Huimin Chen, Zeshu Yu, Zhifeng Ye, Cheng Peng, Ling Xiao, Mi Zhao, Shuisheng Li, Haoran Lin, Yong Zhang

**Affiliations:** ^1^State Key Laboratory of Biocontrol, Guangdong Provincial Key Laboratory for Aquatic Economic Animals and Guangdong Provincial Engineering Technology Research Center for Healthy Breeding of Important Economic Fish, School of Life Sciences, Sun Yat-Sen University, Guangzhou, China; ^2^Guangdong South China Sea Key Laboratory of Aquaculture for Aquatic Economic Animals, Guangdong Ocean University, Zhanjiang, China; ^3^Guangdong Key Laboratory of Animal Conservation and Resource Utilization, Guangdong Public Laboratory of Wild Animal Conservation and Utilization, Guangdong Institute of Applied Biological Resources, Guangzhou, China; ^4^Southern Laboratory of Ocean Science and Engineering (Guangdong, Zhuhai), Zhuhai, China

**Keywords:** FSH, protogynous, sex differentiation, sex steroid hormones, male sex fate

## Abstract

Follicle-stimulating hormone (FSH) signaling is considered to be essential for early gametogenesis in teleosts, but its functional roles during sex differentiation are largely unknown. In this study, we investigated the effects of long-term and short-term FSH injection on sex differentiation in the protogynous orange-spotted grouper (*Epinephelus coioides*). Long-term FSH treatment initially promoted the formation of ovaries but subsequently induced a male fate. The expression of female pathway genes was initially increased but then decreased, whereas the expression of male pathway genes was up-regulated only during long-term FSH treatment. The genes related to the synthesis of sex steroid hormones, as well as serum 11-ketotestosterone and estradiol, were also up-regulated during long-term FSH treatment. Short-term FSH treatment activated genes in the female pathway (especially *cyp19a1a*) at low doses but caused inhibition at high doses. Genes in the male pathway were up-regulated by high concentrations of FSH over the short term. Finally, we found that low, but not high, concentrations of FSH treatment activated *cyp19a1a* promoter activities in human embryonic kidney (HEK) 293 cells. Overall, our data suggested that FSH may induce ovarian differentiation or a change to a male sex fate in the protogynous orange-spotted grouper, and that these processes occurred in an FSH concentration-dependent manner.

## Introduction

It is now well-established that sex steroid hormones play a crucial role during gonadal differentiation in teleost fish (1–3). Moreover, the administration of exogenous steroids in undifferentiated fish can direct the process of sexual differentiation toward a specific sex fate. Treatments with exogenous androgens, or an aromatase inhibitor (AI), can induce a male fate in some fish, regardless of their genetic sex (4–6). Meanwhile, genetic males can be driven into a female fate by estrogen (7, 8). Therefore, the balance of endogenous androgens and estrogens play a crucial role during sex differentiation. However, the upstream mechanisms underlying the synthesis of gonadal sex steroid hormones during sex differentiation are largely unknown.

In teleosts, the synthesis of gonadal sex steroid hormones is largely controlled by two pituitary gonadotropins (GTHs): follicle-stimulating hormone (FSH) and luteinizing hormone (LH) (9, 10). GTHs are formed by a common alpha subunit (CGA) with distinct beta subunits (FSHB and LHB) (11), and function via specific interactions with their respective receptors, the FSH receptor (FSHR) and the LH receptor (LHR) (12). In teleosts, FSH regulates the early phases of gametogenesis, whereas LH is responsible for the final maturational processes of the gametes, such as oocyte maturation, ovulation and spermiation (13–15). For example, loss of *fsh*β resulted in a significant retardation of follicular growth, and a delay in the onset of puberty, while loss of *lh*β led to infertility in female zebrafish (*Danio rerio*), although ovarian growth and the onset of puberty were normal (16). However, FSH treatments induced female-to-male sex change in adult honeycomb grouper (*Epinephelus merra*) (17), suggesting that GTHs exert different functions in different species.

Although the important role of GTHs in gonadal development and maturation in teleost fish is now clearly established, the function of GTHs in sex differentiation remains largely unknown. In fact, FSH has been detected in the pituitary prior to the onset of gonadal sex differentiation in a variety of species (18–20). In the Malabar grouper (*Epinephelus malabaricus*), *fsh*β was present and significantly increased in concentration in the undifferentiated and ovarian differentiation stages, while *lh*β was not expressed before ovarian differentiation and was first detected after ovarian differentiation (21). These reports suggest that FSH may be involved in gonadal sex differentiation.

The orange-spotted grouper, a protogynous hermaphroditic fish of great ecological and economic values, develops ovaries at a young age and then undergoes sex changes to become male at the age of 4–5 years (22). The timing of gonad development can be divided into three stages: Firstly, the primordial gonad appears around 7 weeks after hatching. Secondly, sex differentiation is started around 13–16 weeks after hatching by formation of ovarian cavity and the mitosis of germ cell can be observed at 22–30 weeks after hatching. Thirdly, primary oocyte appears around 34–43 weeks after hatching (23). In our earlier studies, we found that *fsh*β was highly expressed during sex differentiation. Meanwhile, high levels of *fshr* expression were detected during sex differentiation but decreased significantly after sex differentiation. In contrast, *lhr* was not detected during in these two periods (data unpublished). Furthermore, previous study showed that recombinant orange-spotted grouper FSH activating FSH receptor and stimulating *in vitro* testosterone (T) and estradiol-17β (E2) secretion (24). Thus, to investigate the functional roles of FSH on gonadal sex determination in the protogynous orange-spotted grouper, we treated fish with FSH by intraperitoneal injections during sex differentiation, and then analyzed the gonadal phenotype and gene expression profiles. Our results suggest that FSH initially promotes ovarian differentiation in the orange-spotted grouper while a high concentration of FSH may trigger male sex fate.

## Materials and Methods

### Fish

Orange-spotted groupers were obtained ~80 days after hatching (mean weight 5.5 g, body length ~70 mm) or ~130 days after hatching (mean weight 37.5 g, body length ~137.2 mm) and reared in Guangdong Daya Bay Fishery Development Center (Huizhou City, Guangdong, P.R. China). All animal experiments were conducted in accordance with the guidelines and approval of the respective Animal Research and Ethics Committees of Sun Yat-Sen University.

### Hormone Treatment

Short-term and long-term intraperitoneal injections of FSH during sex differentiation were performed. Porcine FSH (Ningbo Sansheng Pharmaceutical Co., Ltd, China) was used in this study since fish FSH was unavailable at the time of the study. FSH was directly dissolved in saline. For the long-term intraperitoneal injection of FSH, fish (~80 days after hatching) were anesthetized with eugenol and given intraperitoneal injection of either saline or FSH-containing saline (100 IU porcine FSH/fish) at weekly intervals for 9 weeks. Fish were then sacrificed and gonadal tissues, blood samples and pituitaries collected at 2, 6, and 10 weeks after treatment. For short-term intraperitoneal injection of FSH, fish (~130 days after hatching) were anesthetized with eugenol and given single intraperitoneal injection of the saline or FSH-containing saline (3 IU, 10 IU, 20 IU, or 100 IU porcine FSH/fish). After treatment, fish were sacrificed and gonadal tissues collected at 3, 6, 12, or 24 h after treatment.

Eleven fish (five for gonadal histology and six for quantitative real-time PCR) and six fish were sacrificed in each group at each sampling time point for long-term and short-term treatments, respectively.

### Gonadal Histology

Gonads were fixed in Bouin's solution overnight at room temperature, dehydrated, and then embedded in paraffin. All tissue blocks were sectioned at 5 μm and stained with hematoxylin and eosin (H&E) for analysis.

### Serum Oestradiol-17β (E2) and 11-Ketotestosterone (11-KT) Assays

Blood samples were collected from the caudal vein of fish from the FSH injection and control group. Serum samples were collected after centrifugation and stored at −20°C. Serum E2 and 11-KT levels were measured using EIA Assay kits (Cayman Chemical Co, USA) in accordance with the manufacturer's instructions.

### RNA Isolation, Reverse Transcription, and Quantitative Real-Time PCR

Total RNA was isolated by TRIzol and reverse transcribed using a Transcriptor First Strand cDNA Synthesis Kit (Roche, Switzerland) in accordance with the manufacturer's instructions. Quantitative real-time PCR (qPCR) analyses were performed on a Roche Light-Cycler 480 real time PCR system using SYBR Green I Master Mix (Roche) according to the manufacturer's protocol. The real-time qPCR conditions were as follows: denaturation at 95°C for 10 min, followed by 40 cycles of 95°C for 10 s, 55°C for 20 s, and 72°C for 20 s. The mRNA levels of *cyp19a1a, cyp11b, foxl2, dmrt1, sox9, fshr, star, cyp11a1, cyp17a1, hsd3b*, and *hsd17b* were then analyzed with β-actin serving as an internal control. After amplification, the fluorescent data were converted to threshold cycle values (Ct). The relative abundance of mRNA transcripts was then evaluated using the formula: *R* = 2^−ΔΔ*Ct*^, as described previously (25). The primers used in this study are listed in Table 1.

**Table 1:** Primers used in this study.

**Primers**	**Primers Sequence (from 5^**′**^ to 3^**′**^)**
**Primers for real-time PCR**	
q*cyp19a1a*-F	GGAGACATTGTGAGAGTCTGGATC
q*cyp19a1a*-R	GACAGGTACATCCAGGAAGA
q*cyp11b*-F	TGTTGCCGTCTGACATCG
q*cyp11b*-R	TCGCCACTCCTCACCGTTC
q*dmrt1*-F	GCTGGAGTAGACTGCTTGTTT
q*dmrt1*-R	CGACTGTGCGTCAGTATGAGC
q*sox9*-F	GCAATGCAGGCTCAGAATAG
q*sox9*-R	GGTATCAAGGCAGTACCCAG
q*foxl2*-F	CCACCGTACTCCTATGTCGC
q*foxl2*-R	GTCTGATACTGTTCTGCCAAC
q*star*-F	AAGCTCCCTCCTTAGTTCTC
q*star*-R	TTGCTCTAGCATCACCTCC
q*hsd17b-F*	GGTTGCGTGACAGTGTTCTT
q*hsd17b-R*	TTTGTTCCCGCTTGCATGAA
q*cyp11a1-F*	CTGAAGTAGTGTGACTCCGTCCTTAAC
q*cyp11a1-R*	GGCAGAGACCCCAAAGTGTTC
q*cyp17a1-F*	GGGATTTCACAGTGAGAAAAGGA
q*cyp17a1-R*	CAAAGAGCTCAGGGTTTTTCCA
q*hsd3b*-F	CTGGAGGACTGTAGAGGCG
q*hsd3b*-R	GGTGCTGGTGTAGATGAAGG
q*β-actin*-F	ACCATCGGCAATGAGAGGTT
q*β-actin*-R	ACATCTGCTGGAAGGTGGAC
*qfshr*-F	CGAGGCTGACCCTTACTTCC
*qfshr*-R	GATCCAGATGAGGACCCGTA
**Primers for cloning**	
*gfshr-*F	CTGGCTAGCATGATGATGATAATGATTG
*gfshr-*R	CGGAATTCGCAGTCTTGTTTCCCATC
*gfoxl2*-F	CAGTGTGGTGGAATTGTGCGCAATGATGGCCAC
*gfoxl2*-R	ATATCTGCAGAATTGGTAAATTTTAAATATCAATCCTCGTGTGTA
*gdmrt1*-F	CGGGGTACCGCCATGAGCAAAGATAAGCAGAG
*gdmrt1*-R	CCGGAATTCCGTTTTATTCATTTGGTGGCG
*gcyp19a1a* promoter-F	CGGGGTACCGAGGAGTTGATAAATTCTGTTCCGAC
*gcyp19a1a* promoter-R	CCGCTCGAGCACAAGCAGAGATGAGATCCATAAGAA

### Immunohistochemistry (IHC)

Rabbit anti-Dmrt1 antibody (polyclonal) was produced by our laboratory and IHC analyses were performed as described previously (26). Antibodies against DMRT1 were diluted at a ratio of 1:100. The HRP-conjugated Goat Anti-Rabbit/Mouse IgG (H+L) (Proteintech, USA) was used as secondary antibody and positive signals were detected by DAB staining. The sections were counterstained with hematoxylin after IHC staining. Photographic images of the samples were taken under a Nikon light microscope (Japan).

### Dual-Luciferase Assay

In order to generate pcDNA-gDMRT1, pcDNA-gFOXL2, and pcDNA-gFSHR plasmids, the complete open reading frames (ORFs) of *gdmrt1, gfoxl2*, and *gfshr* (GenBank Accession Nos. EF017802.1, JQ178341.1 and HQ650769.1, respectively) were amplified by PCR, using the high-fidelity KOD Plus polymerase (Toyobo, Japan) and then subcloned into the pcDNA4 TO myc-His A expression vector (Invitrogen, USA). The putative promoter regions of *cyp19a1a* (GenBank Accession No JF420889) were then inserted upstream of the Firefly luciferase gene of the pGL4.10-basic vector (Promega, USA) to generate reporter plasmids. Human embryonic kidney (HEK) 293T cells (3111C0001CCC000091, National Infrastructure of Cell Line Resource, China) were grown in DMEM (Hyclone, USA) supplemented with 10% fetal bovine serum (Gibco, USA) under 5% CO_2_ at 37°C. Cells were then cultured in 48-well plates (1 × 10^5^ cells/well) and transfections (70–90% confluent at transfection) were performed using Lipofectamine 2000 reagent (Invitrogen, USA) with the following plasmids: (1) 100 ng/well of the pGL4-Cyp19a1a promoter luciferase reporter vector; (2) 0–150 ng/well pcDNA4 expression plasmid (Invitrogen, USA), containing the cDNAs encoding gfshr, gfoxl2 and gdmrt1, and (3) 10 ng/well of pRL-TK vector (Promega, USA). The total amount of transfected plasmid was adjusted to 260 ng/well with empty vectors. Renilla luciferase from pRL-TK was used as an internal control for transfection efficiency. Approximately 6 h after transfection, the medium of cells which were transfected with the g*foxl2* and g*dmrt1* expression plasmid was aspirated and replaced with medium containing 5% fetal bovine serum. For cells transfected with g*fshr*, fresh medium supplemented with 5% fetal bovine serum and FSH (0–25 IU) was applied. Luciferase activity was measured 30 h after transfection using the Dual-Luciferase Reporter Assay System (Promega, USA), according to the manufacturer's instructions.

### Statistical Analysis

Quantitative data were expressed as the mean ± standard error of the mean (SEM). Significant differences were identified by Independent-Samples T-Test, one-way or two-way analysis of variance (ANOVA) using SPSS and GraphPad Prism 6. A probability level which was equal to or < 0.05 indicated a statistically significant difference.

## Results

### FSH Injection Accelerated Gonad Development and Induced Female-to-Male Sex Fate Change

**Figure 1:**
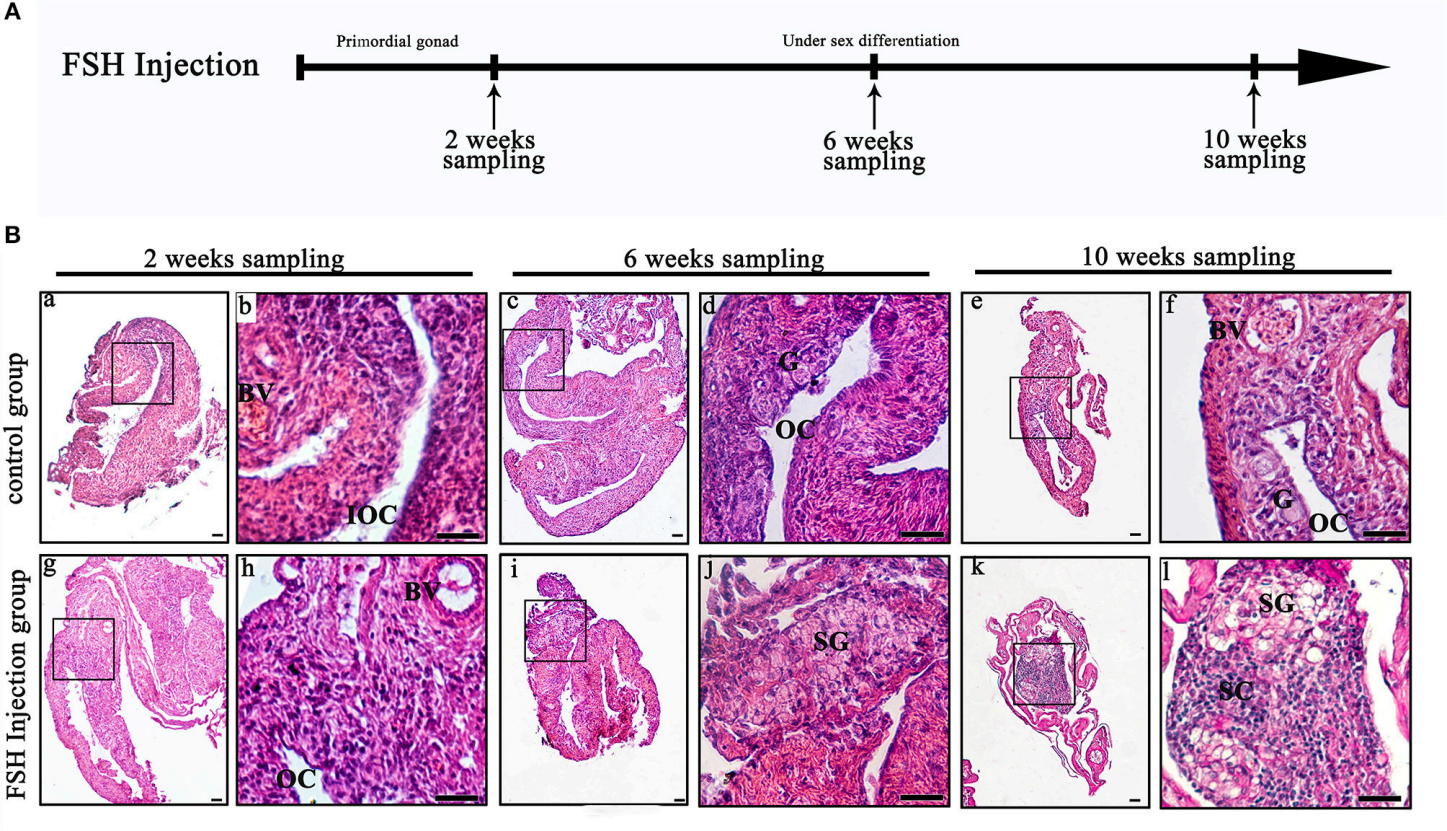
Gonad histology during long-term FSH injection. **(A)** The time schedule of sampling is indicated by arrows. **(B)** Gonadal histology. (a–f) Gonadal histology of the control group; (g–l) Gonadal histology of the FSH injection group; (b,d,f,h,j,l) Represent magnified views of the boxed areas in (a,c,e,g,i,k). BV, blood vessel; IOC, initial ovarian cavity; OC, ovarian cavity; G, germ cell; SG, spermatogonia; SC, spermatocytes; scale bars, 25 μm.

To investigate the function of FSH on sexual differentiation in the orange-spotted grouper, we selected fish around 80 days after hatching (i.e., with a undeveloped gonad) and administered intraperitoneal injection of either saline or FSH-containing saline (100 IU porcine FSH/fish) at weekly intervals for 9 weeks. Then, the gonads of the control group and the FSH injection group were sampled from 2 to 10 weeks after treatment (Figure 1A). In the controls, the gonads developed, and showed an ovarian cavity at 2 weeks after treatment, with germ cells appearing at 6 and 10 weeks after treatment (Figures 1Ba–f). In the long-term FSH injection group, gonad development was accelerated and a complete ovarian cavity was formed by week 2 of treatment (Figures 1Bg,h); however, this ovarian cavity was reversed into testes at 6 and 10 weeks after treatment (Figures 1Bi–l). The ovarian cavity disappeared and a number of spermatogonia appeared 6 weeks after treatment. After 10 weeks of treatment, the gonads had become testes and were full of spermatogonia and spermatocytes. Collectively, these data demonstrate that FSH can accelerate gonad development and induce female-to-male sex fate change during sex differentiation (Table 2).

**Table 2:** The gonadal status of *Epinephelus coioides* during experimental period.

**Duration of administration**	**Sample No**	**Gonadal status**					
		**Ovary^a^**	**Ovary^b^**	**Ovary^c^**	**Testis^d^**	**Testis^e^**	**Testis^f^**
Initial control at 80 day after hatching	8	8	0	0	0	0	0
**Control group**						
2 weeks	5	4	1	0	0	0	0
6 weeks	5	1	3	1	0	0	0
10 weeks	5	0	1	4	0	0	0
**Long-term FSH injection group**						
2 weeks	5	2	3	0	0	0	0
6 weeks	5	1	0	0	1	3	0
10 weeks	5	0	0	0	0	2	3

a *Ovary: contain initial ovarian cavity*.

b *Ovary: contain ovarian cavity*.

c *Ovary: contain ovarian cavity and germ cell*.

d *Testis: contain efferent duct and there are not many male germ cells*.

e *Testis: contain spermatogonia*.

f *Testis: contain spermatogonia and spermatocytes*.

### Detection of Male Germ Cells During FSH Injection

**Figure 2:**
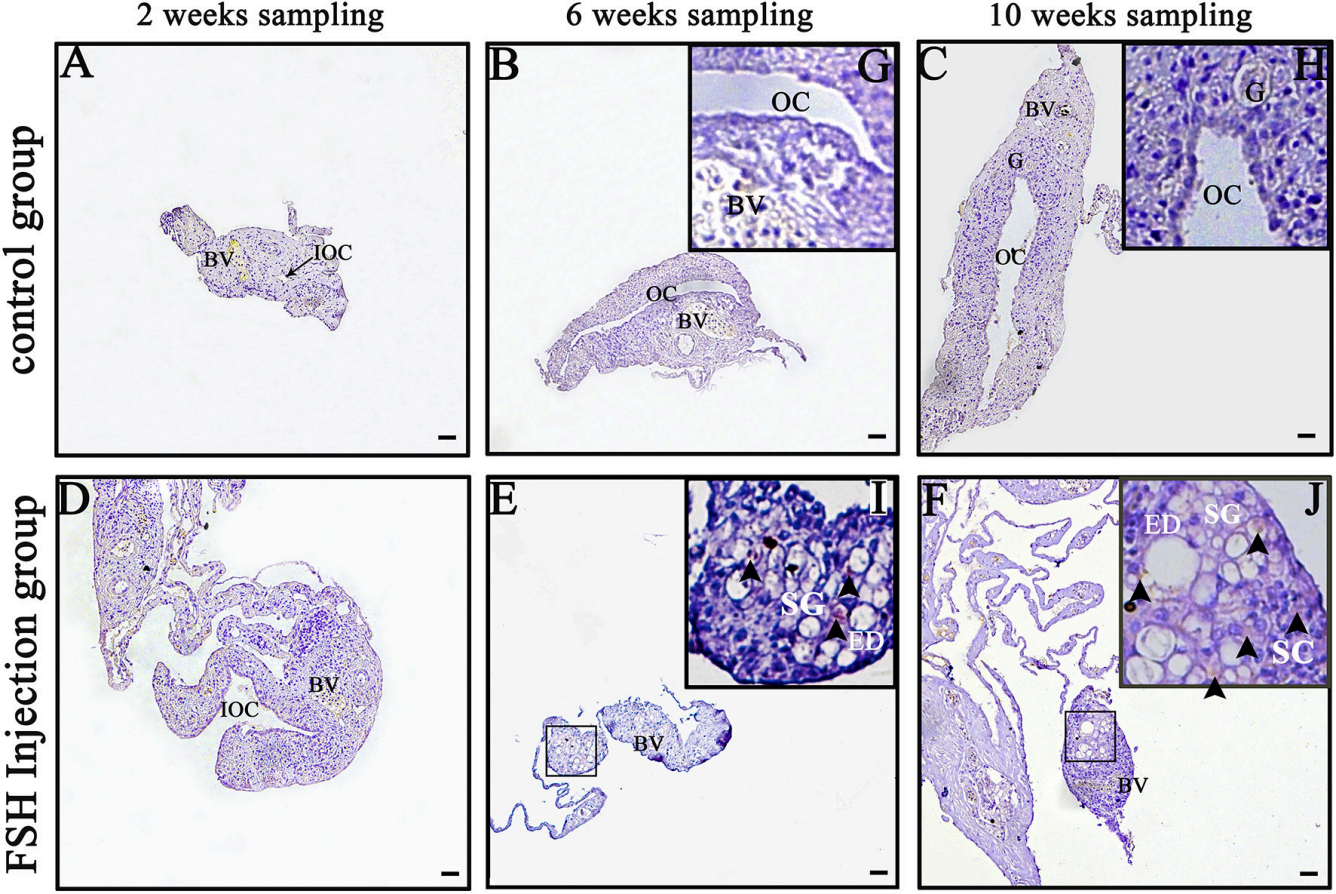
Detection of DMRT1 signals during long-term FSH injection. Results of immunohistochemistry using an anti-DMRT1 antibody in gonads at different sampling times; **(A–C)** The control group; **(D–F)** the FSH injection group; **(G–J)** show high magnification images of the boxed area in **(B,C,E,F)**, respectively. The black arrowheads indicate positive signals. BV, blood vessel; ED, efferent duct; OC, ovarian cavity; G, germ cell; SG, spermatogonia; SC, spermatocytes. Scale bars, 25 μm.

*Dmrt1* is a male-specific marker in the orange-spotted grouper because its protein is only detected in spermatogonia and spermatocytes (27). In order to distinguish between spermatogonia and oogonia, we examined DMRT1 protein using IHC during FSH injection studies. In the control group, no DMRT1-positive signals were detected (Figures 2A–C,G,H). In the long-term FSH injection group, DMRT1-positive signals were detected in spermatogonia around the efferent duct 6 weeks after treatment and spermatocytes appeared 10 weeks after treatment (Figures 2D–F,I,J). These results suggested that long-term FSH injection during sex differentiation can induce female-to-male sex change.

### Gene Expression Profiles and Serum Steroid Hormone Levels During FSH-Induced Sex Fate Change

**Figure 3:**
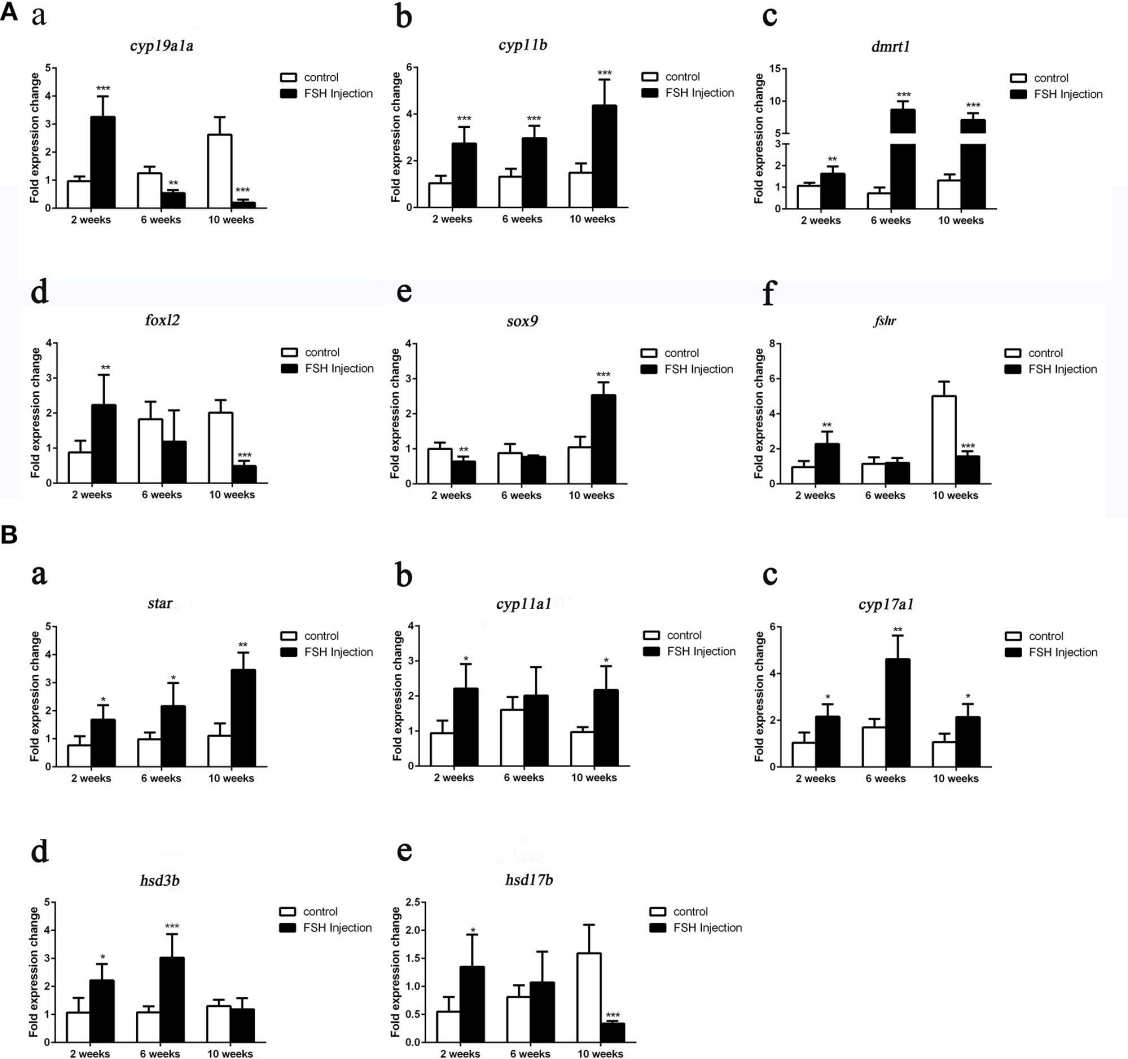
Expression profiles of key genes related to sex differentiation and sex steroid hormones synthesis during long-term FSH injection. **(A)** Gene expression of key genes related to sex differentiation: *cyp19a1a* (a), *cyp11b* (b), *dmrt1* (c), *foxl2* (d) *sox9* (e), and *fshr* (f) was analyzed by real-time qPCR. **(B)** Expression of genes related to sex steroid hormones synthesis: *star* (a), *cyp11a1* (b), *cyp17a1* (c)*, hsd3b* (d), and *hsd17b* (e) was analyzed by real-time qPCR. β-actin was used as an internal control. Data are expressed as the mean ± SEM (*n* = 3–5). ^*^P < 0.05, ^**^P < 0.01, and ^***^P < 0.001 between the control group and the FSH injection group.

**Figure 4:**
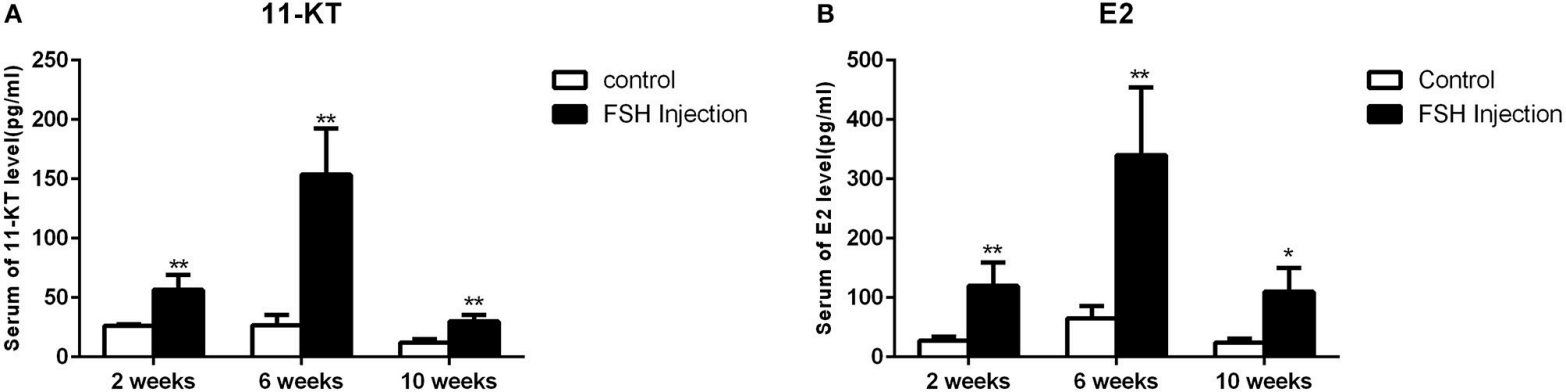
Serum sex steroid hormone level. **(A)** Levels of 11-KT in the control and FSH injection group. **(B)** Levels of E2 in the control and FSH injection group. Data are expressed as the mean ± SEM (*n* = 4–6). ^*^P < 0.05, and ^**^P < 0.01 between the control group and the FSH injection group.

Next, we analyzed the effects of long-term FSH injection on the expression of key genes related to sex differentiation. The expression of both *cyp19a1a* and *foxl2* was significantly increased 2 weeks after treatment but significantly decreased by 6 and 10 weeks after treatment, respectively (Figures 3Aa,d). In contrast, the expression of *cyp11b* and *dmrt1* was significantly increased approximately 4 times and 10 times at all sampling time points, respectively (Figures 3Ab,c). Expression levels of *sox9* were significantly decreased 2 weeks after treatment but significantly increased 2.5-folds at 10 weeks after treatment (Figure 3Ae). The expression of *fshr* was up-regulated at 2 weeks after treatment but significantly down-regulated at 10 weeks after treatment (Figure 3Af). In conclusion, long-term FSH injection first stimulated gene expression, both in the male and female pathway, but then suppressed gene expression in the female pathway. In teleosts, the synthesis of gonadal sex steroid hormones is controlled by gonadotropins. Therefore, the expression profiles of key genes related to the synthesis of gonadal sex steroid hormones were also analyzed. The expression of *star* and *cyp17a1* were significantly increased at all sampling time points (Figures 3Ba,c). Similarly, the expression of *cyp11a1* was significantly increased 2 and 10 weeks after treatment, while the expression of *hsd3b* was significantly increased 2 and 6 weeks after treatment (Figures 3Bb,d). However, the expression of *hsd17b* was up-regulated 2 weeks after treatment but significantly down-regulated 10 weeks after treatment (Figure 3Be). Consistent with the mRNA level of key genes related to the synthesis of gonadal sex steroid hormones, the serum levels of 11-KT and E2 were significantly increased at all sampling time points (Figures 4A,B). In general, long-term FSH injection stimulated the synthesis of gonadal sex steroid hormones.

### Gene Expression Profiles During Short-Term FSH Injection

**Figure 5:**
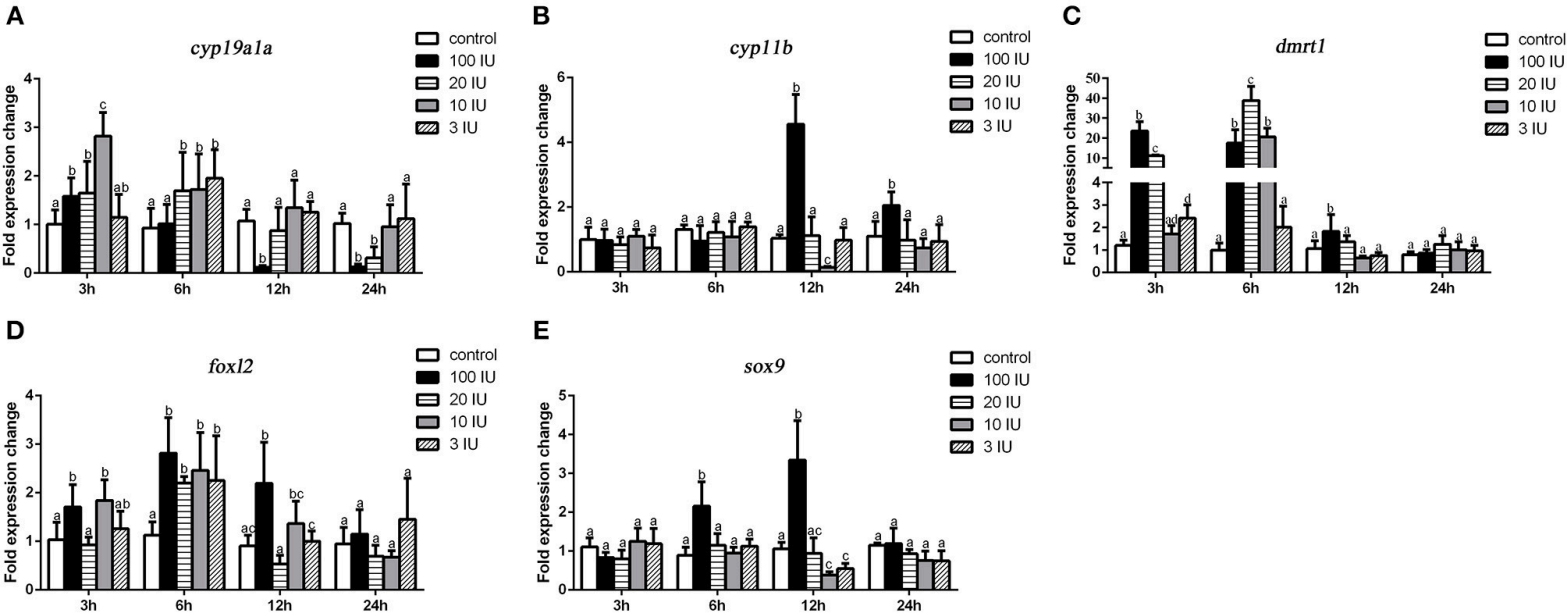
Expression profiles of key genes related to sex differentiation in the gonads after short-term FSH injection. Gene expression of *cyp19a1a* **(A)**, *cyp11b* **(B)**, *dmrt1* **(C)**, *foxl2* **(D)**, and *sox9* **(E)** was analyzed by real-time qPCR. β*-actin* was used as an internal control to determine relative gene expression. Data are expressed as the mean ± SEM (*n* = 4–6). Different letters represent statistically significant differences (*P* < 0.05).

We further analyzed the short-term effects of different doses of single injection of FSH on the expression of genes related to sex differentiation. Gonadal tissues were collected at 3, 6, 12, and 24 h after treatment, and the mRNA levels of key genes related to sex differentiation were examined. Compared to the controls, the expression of *cyp19a1a* was significantly up-regulated at all concentrations of FSH 3 or 6 h after treatment, but significantly down-regulated 12 and 24 h after treatment in the 20 IU and 100 IU FSH injection groups (Figure 5A). Similarly, the expression of *foxl2* was significantly up-regulated at all concentrations 3 or 6 h after treatment, but only down-regulated 12 h after treatment in the 20 IU FSH injection group (Figure 5D). The expression of *cyp11b* was up-regulated in the 100 IU FSH injection group only at 12 and 24 h after treatment and was suppressed in the 10 IU FSH injection group 12 h after treatment (Figure 5B). Similarly, the expression of *sox9* was significantly up-regulated in the 100 IU FSH injection group at 6 and 12 h after treatment and suppressed in the 3 IU and 10 IU FSH injection groups 12 h after treatment (Figure 5E). The expression of *dmrt1* was significantly up-regulated in all FSH concentrations 3 or 6 h after treatment and up-regulated in the 100 IU FSH injection group only 12 h after treatment (Figure 5C). In conclusion, FSH activated the expression of female pathway genes at low concentrations but suppressed expression of these genes at high concentrations. Furthermore, a high dose of FSH induced the expression of male pathway genes.

### The Effect of FSH/FSHR Signaling, FOXL2, and DMRT1 on cyp19a1a Promoter Activity

**Figure 6:**
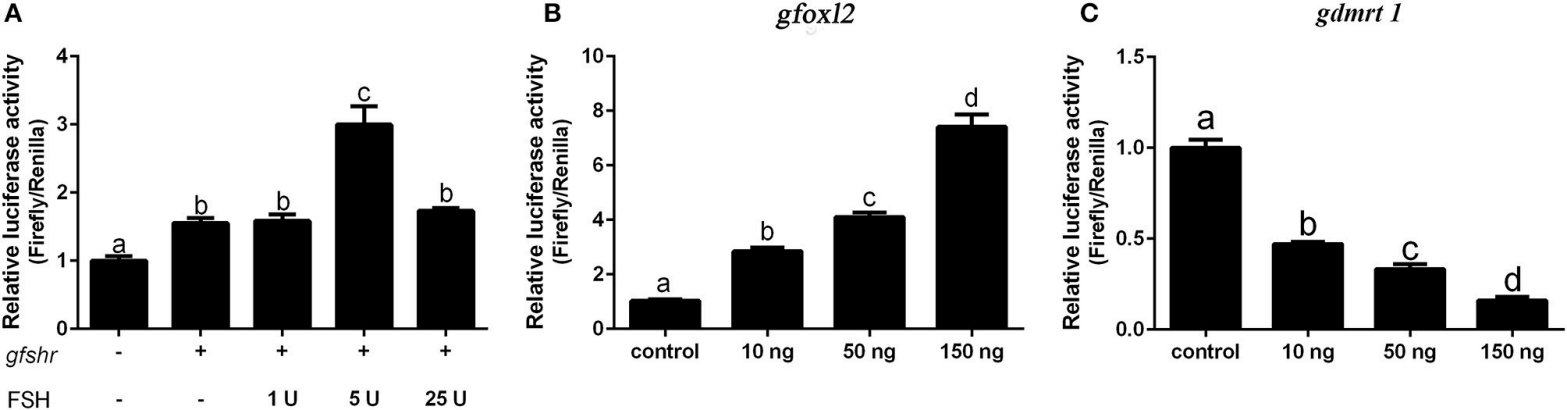
The effect of FSH/FSHR signaling, FOXL2 and DMRT1 upon *cyp19a1a* promoter activity in HEK 293T cells. **(A)** The effect of different FSH (1–25 IU) concentrations upon *cyp19a1a* promoter activity. **(B,C)** The effect of different *gfoxl2* and *gdmrt1* expression vectors (10–150 ng/well) on *cyp19a1a* promoter activity. Data are expressed as the mean ± SEM (*n* = 4~6). +, 150 ng/well. Different letters represent statistically significant differences (*P* < 0.05).

We next examined whether grouper *cyp19a1a* gene transcription is regulated by FHS/FSHR signaling, *foxl2*, or *dmrt1* in HEK 293T cells. The results showed that DMRT1 suppressed activity while FOXL2 activated *cyp19a1a* expression in a dose-dependent (10–150 ng) manner (Figures 6B,C). A low concentration of FSH (5 IU), but not high concentrations of FSH (25 IU), significantly stimulated *cyp19a1a* gene transcription (Figure 6A), suggesting that FSH may stimulate *cyp19a1a* gene transcription at low concentrations but suppressed it at a high concentration.

## Discussion

In teleosts, FSH plays an important role in reproductive activities, such as oogenesis in females and spermatogenesis in males, but its role in sex differentiation is not clear. In this study, we discovered that long-term FSH treatment firstly accelerates ovarian differentiation but subsequently induces female-to-male sex reversal in orange-spotted grouper during sex differentiation. Interestingly, treating adult honeycomb grouper (*E. merra*) females with bovine FSH (50 or 500 ng/fish) or juvenile tiger grouper (*Epinephelus fuscoguttatus*) with recombinant giant grouper FSH (200 μg/kg body weight) induced female-to-male sex change (17, 28). The expression of *fsh*β was low in females but increased significantly during female-to-male sex reversal in the honeycomb grouper. Similar *fsh*β expression profiles were reported in the orange-spotted grouper and the long-tooth grouper (*Epinephelus bruneus*) during female-to-male sex reversal induced by 17α-methyltestosterone (MT) and fadrozole (aromatase inhibitor), respectively (29, 30). We also detected the *fshr* mRNA level and the expression of *fshr* was up-regulated at female stage and suppressed at male stage. Previous study in the orange-spotted grouper show that the expression of *fshr* was significantly inhibited throughout the MT-induced sex change process (31). These data suggested that FSH is involved in female-to-male sex reversal and that a high concentration of FSH is required to trigger the male fate in this protogynous grouper.

To understand the mechanism of FSH-induced change in female-to-male sex fate, we then examined the mRNA levels of sex-related genes. The expression of *cyp19a1a* and *foxl2* were initially increased but then decreased significantly, whereas the expression of *cyp11b, dmrt1*, and *sox9* were significantly up-regulated after long-term FSH treatment. The expression of *cyp19a1a* is associated with sex differentiation and sex fate change. In tilapia (*Oreochromis niloticus*) and rice field eel (*Monopterus albus*), the *cyp19a1a* gene was up-regulated during the formation and maintenance of ovaries, but down-regulated during female-to-male sex reversal (32, 33). The transcriptional suppressors of *cyp19a1a* (*dmrt1* and *sox9*) are required for testicular differentiation whereas the transcriptional activator of *cyp19a1a* (*foxl2*) is needed for ovarian differentiation (34–36). Therefore, long-term FSH treatment firstly activated the expression of female pathway genes and accelerates the formation of ovarian cavity. However, the continuous treatment with FSH suppressed the expression of female pathway genes and activated the expression of male pathway genes, leading to a male fate. Moreover, we analyzed the mRNA level of genes related to sex steroid hormones synthesis. All genes tested were up-regulated after FSH injection except for *hsd17b* which decreased 10 weeks after treatment. Consistent with the gene expression profiles, the serum levels of 11-KT and E2 were significantly increased compared to the control fish, but decreased after 10 weeks compared to the 6-week timepoint in the treatment group. Similarly, recombinant or affinity-purified FSH significantly enhanced the synthesis of 11-KT and E2 in tilapia and rainbow trout (*Oncorhynchus mykiss*) (37, 38). It therefore appears that treatment with high doses of FSH during sex differentiation increased endogenous 11-KT and E2 levels but suppressed the expression of *cyp19a1a*, thus leading to male fate. This hypothesis is in agreement with our previous study (23). Treating fish with E2 and MT simultaneously during sex differentiation resulted in female-to-male sex reversal. Meanwhile, the expression of *cyp19a1a* was suppressed and the serum levels of 11-KT and E2 were also increased. In fact, the serum E2 level in the FSH treatment group was unexpected. The expression of *cyp19a1a* was significantly decreased 6 and 10 weeks after treatment, but the serum E2 level increased. *cyp19a1a* encodes aromatase, an enzyme which catalyzes the synthesis of estrogen. One possible explanation for these findings is that FSH treatment stimulates the production of sex steroid hormones and significantly elevates the concentration of testosterone, the precursor of E2. Another possibility explanation is the slowing down of clearance rate of aromatase.

We further detected DMRT1-expressing cells by immunohistochemistry to identify male germ cells during FSH-induced female-to-male sex reversal. DMRT1-positive signals were firstly detected in spermatogonia around the efferent duct; later, spermatocytes were evident. These results were similar to our previous study in which sex reversal was induced by MT or MT+E2 (39). In MT- or [MT+E2]-induced female-to-male sex reversal, the DMRT1-positive signals were firstly detected in cells around the efferent duct, and then spermatogonia and spermatocytes became evident. In summary, long-term FSH induced female-to-male sex reversal in orange-spotted grouper and new testicular tissues developed from the preformed ovarian tissues.

Although female-to-male sex fate change was observed at the end of long-term FSH treatment, it first accelerated ovarian differentiation. Similar result was observed in seven band grouper, exogenous FSH treatment stimulate ovarian growth in the post-spawning period (40). In red-spotted grouper (*Epinephelus akaara*) and Malabar grouper (*E. malabaricus*), *fsh*β was expressed before the onset of sex differentiation (21, 41). In addition, the expression of *cyp19a1a* was up-regulated after 2 weeks of FSH treatment. It is known that *cyp19a1a* plays a central role in sex differentiation (42, 43). In zebrafish, *cyp19a1a* is expressed before sex differentiation and the down-regulation of *cyp19a1a* expression is likely to be responsible for testicular differentiation (44, 45). These data indicated that FSH is involved in the ovarian differentiation of orange-spotted grouper by stimulating the expression of *cyp19a1a*. Furthermore, the bioactivity of FSH may be associated with its concentration.

In order to clarify the effect of FSH/FSHR signaling on the process of female-to-male sex reversal, we first examined the effect of different concentrations of FSH (3–100 IU/fish) on the expression of sex-related genes. The expression of *cyp19a1a* and *foxl2* were significantly up-regulated in all treatment groups but subsequently decreased in the high concentration treatment groups (20 and 100 IU/fish). In contrast, the expression of *cyp11b* and *sox9* was increased in the high concentration treatment groups (100 IU/fish) but down-regulated in the low concentration treatment groups (3 and 10 IU/fish). Furthermore, the expression of *dmrt1* was increased in the high concentration treatment groups. Therefore, FSH activates female pathway gene expression at low concentrations but suppresses it at high concentrations. In addition, the transcription factors genes, *foxl2* and *dmrt1*, are the key regulators of *cyp19a1a*. Therefore, we examined how FHS/FSHR signaling genes, *foxl2* or *dmrt1*, regulated *cyp19a1a* gene transcription in grouper *in vitro*. The results showed that DMRT1 suppressed *cyp19a1a* expression while FOXL2 activated its expression in a dose-dependent (10–150 ng) manner. Similar results had been reported in other fish species (20, 36, 46). Interestingly, FSH activates *cyp19a1a* expression at a low dose (5 IU) but not at a high dose (25 IU). Accordingly, low concentrations of FSH activate the expression of *cyp19a1a* to promote ovarian differentiation but suppress expression at high concentrations. Furthermore, high concentrations of FSH are needed to trigger the male differentiation pathway in the orange-spotted grouper.

**Figure 7:**
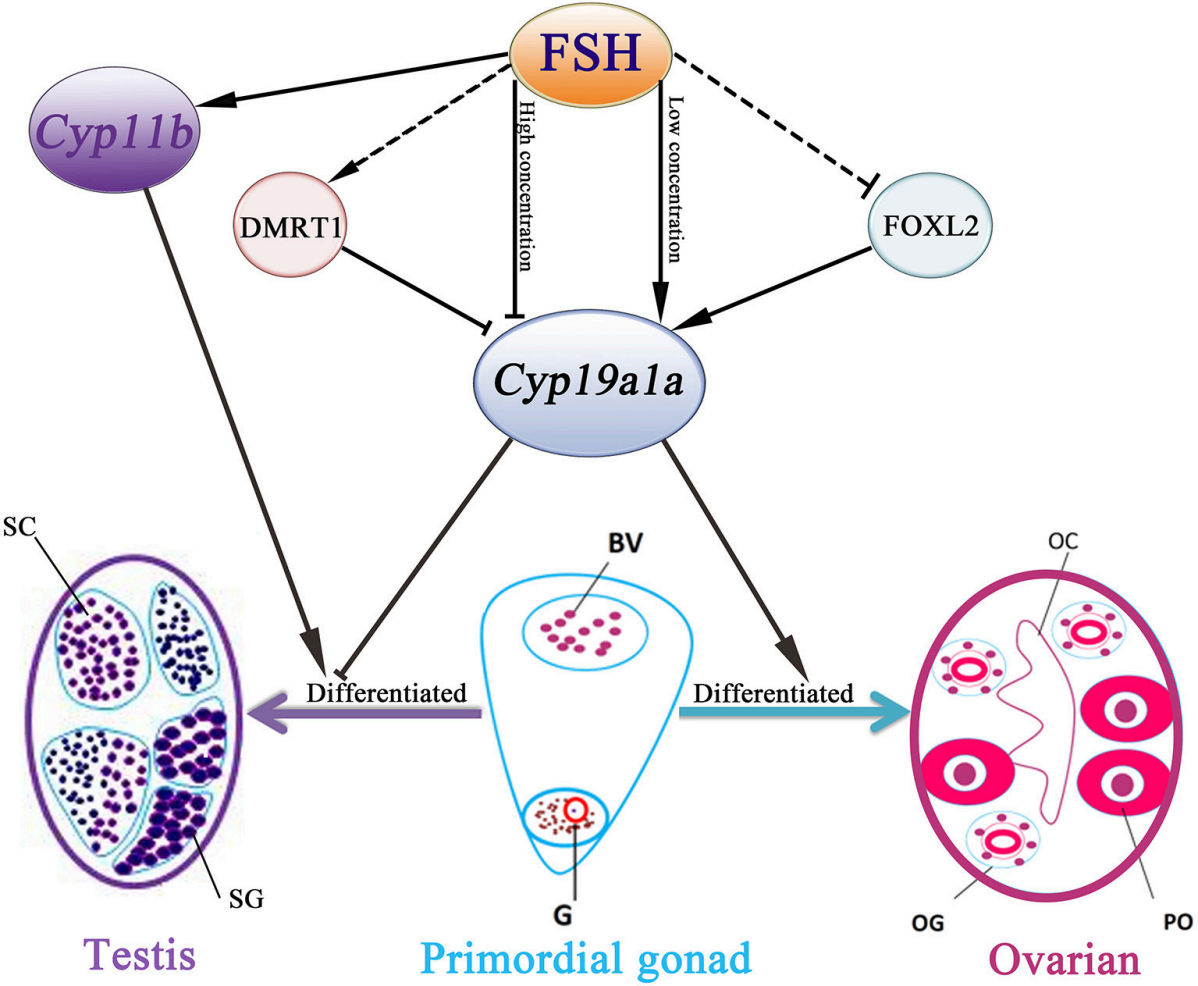
Schematic representation of FSH bioactivity on sex differentiation in the orange-spotted grouper. FSH may function in a concentration-dependent manner in the orange-spotted grouper: promote the process of ovarian differentiation in low concentration or induce a male fate in high concentration. BV, blood vessel; OC, ovarian cavity; OG, oogonium; G, germ cell; PO, primary oocyte; SG, spermatogonia; SC, spermatocytes.

In conclusion, our data demonstrated that FSH is involved in sex differentiation and female-to-male sex fate change in the protogynous orange-spotted grouper (Figure 7). Specifically, FSH may function in a concentration-dependent manner, with low doses of FSH associated with the active synthesis of sex steroid hormones during ovary development and female stages while a high dose of FSH suppressed the expression of female pathway genes (especially *cyp19a1a*) to induce a male fate. Our research provides insight into the sex control of orange-spotted grouper in aquaculture.

## Data Availability

All datasets generated for this study are included in the manuscript and/or the supplementary files.

## Ethics Statement

All animal experiments were conducted in accordance with the guidelines and approval of the respective Animal Research and Ethics Committees of Sun Yat-Sen University.

## Author Contributions

MH, JC, HC, ZSY, ZFY, and CP conducted all the experiments and analyzed the data. LX, MZ, SL, HL and YZ designed the experiments. MH and YL wrote the manuscript.

### Conflict of Interest Statement

The authors declare that the research was conducted in the absence of any commercial or financial relationships that could be construed as a potential conflict of interest.
